# Supplementary figures and images for: Microbial invasion of a toxic medium is facilitated by a resident community but inhibited as the community co-evolves
Source: ISME J. 2022 Sep 14;16(12):2644–52. doi: 10.1038/s41396-022-01314-8 (PMC9666444; doi:10.1038/s41396-022-01314-8)

**A****4-species community**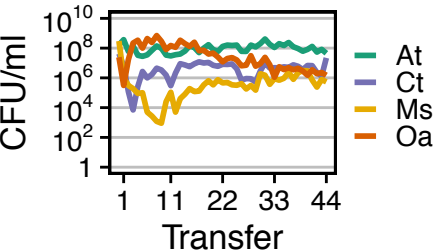**B****3-species community**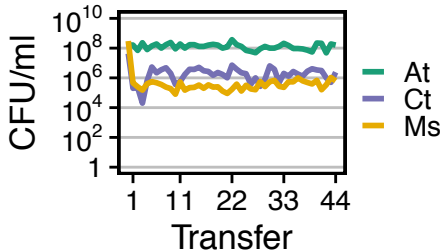

Supplement: Supplementary file 2 — Figure S1 [file 41396_2022_1314_MOESM2_ESM.pdf]

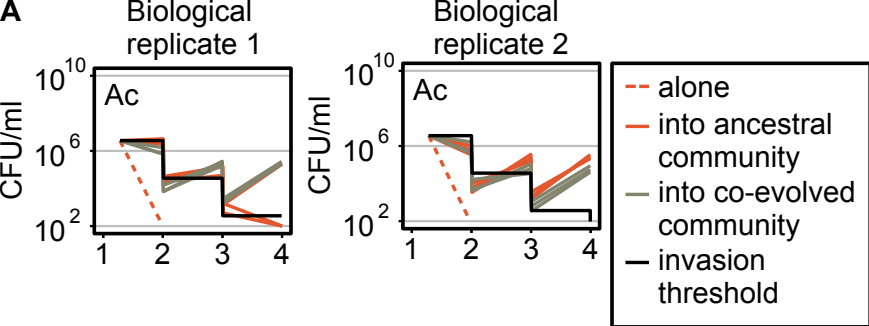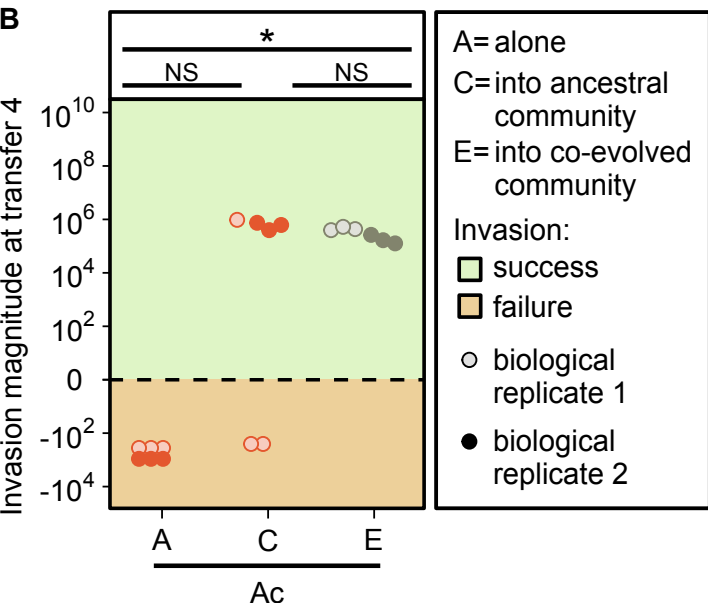

Supplement: Supplementary file 3 — Figure S2 [file 41396_2022_1314_MOESM3_ESM.pdf]

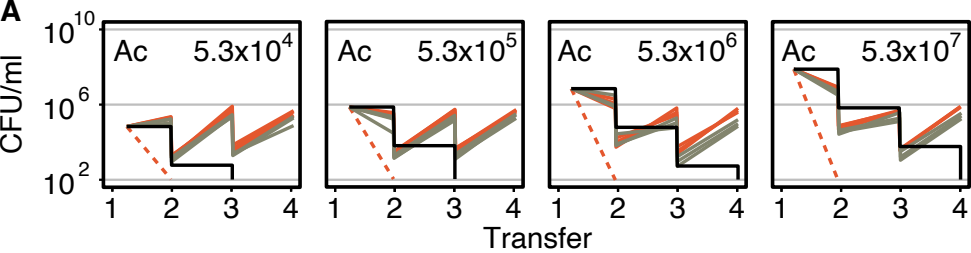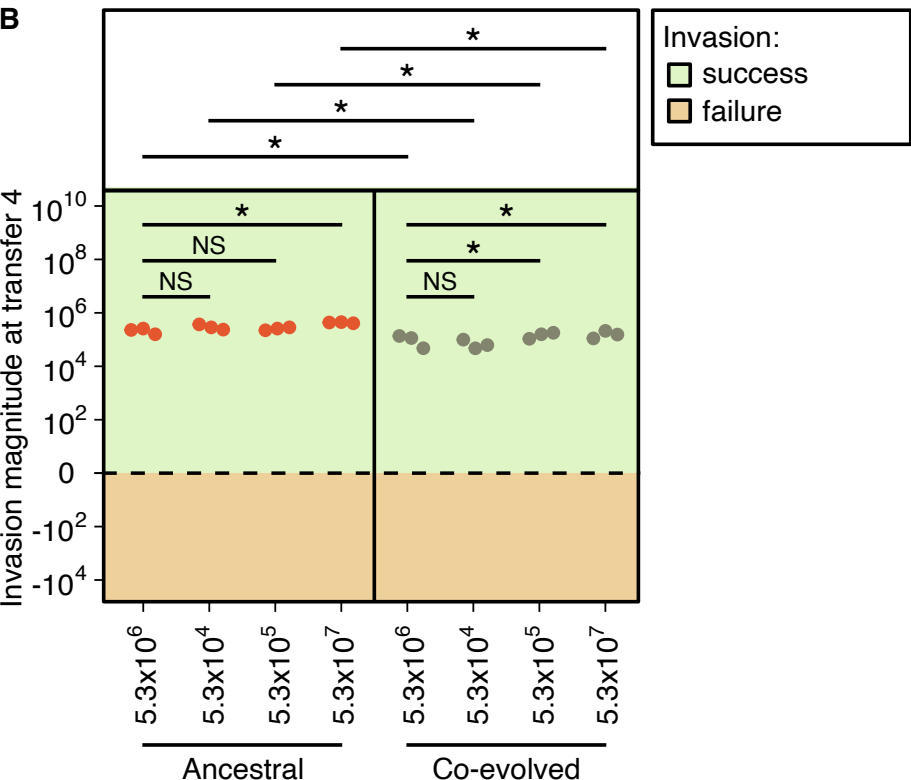

Supplement: Supplementary file 4 — Figure S3 [file 41396_2022_1314_MOESM4_ESM.pdf]

**A**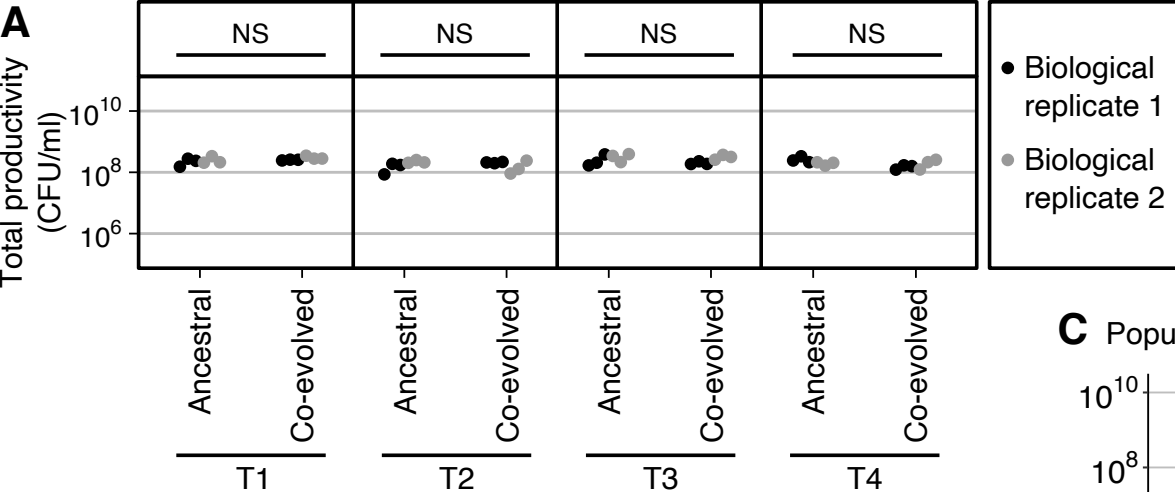**B**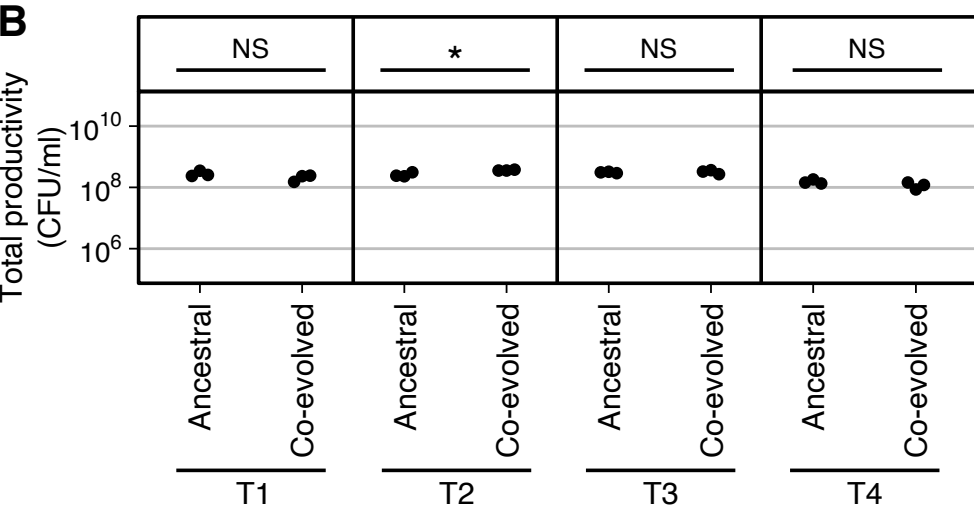**C** Population size at invasion time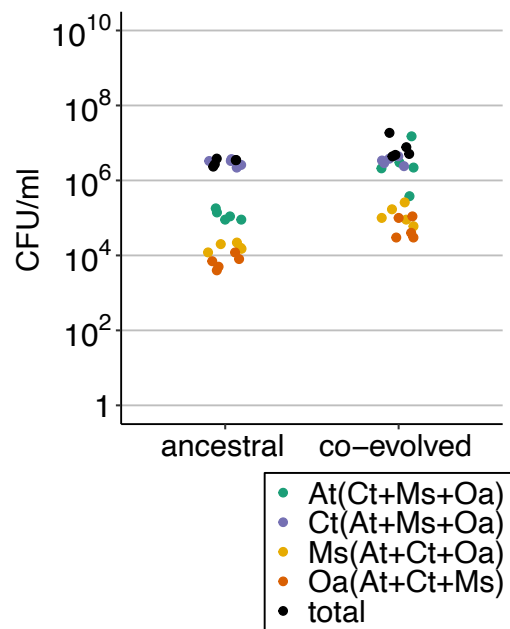

Supplement: Supplementary file 5 — Figure S4 [file 41396_2022_1314_MOESM5_ESM.pdf]

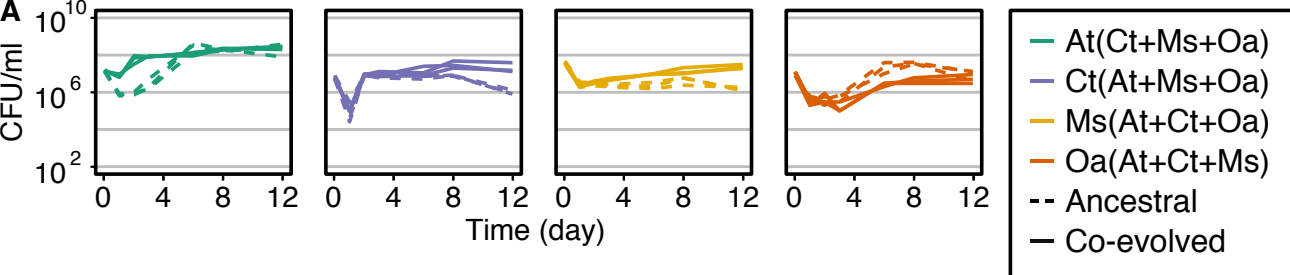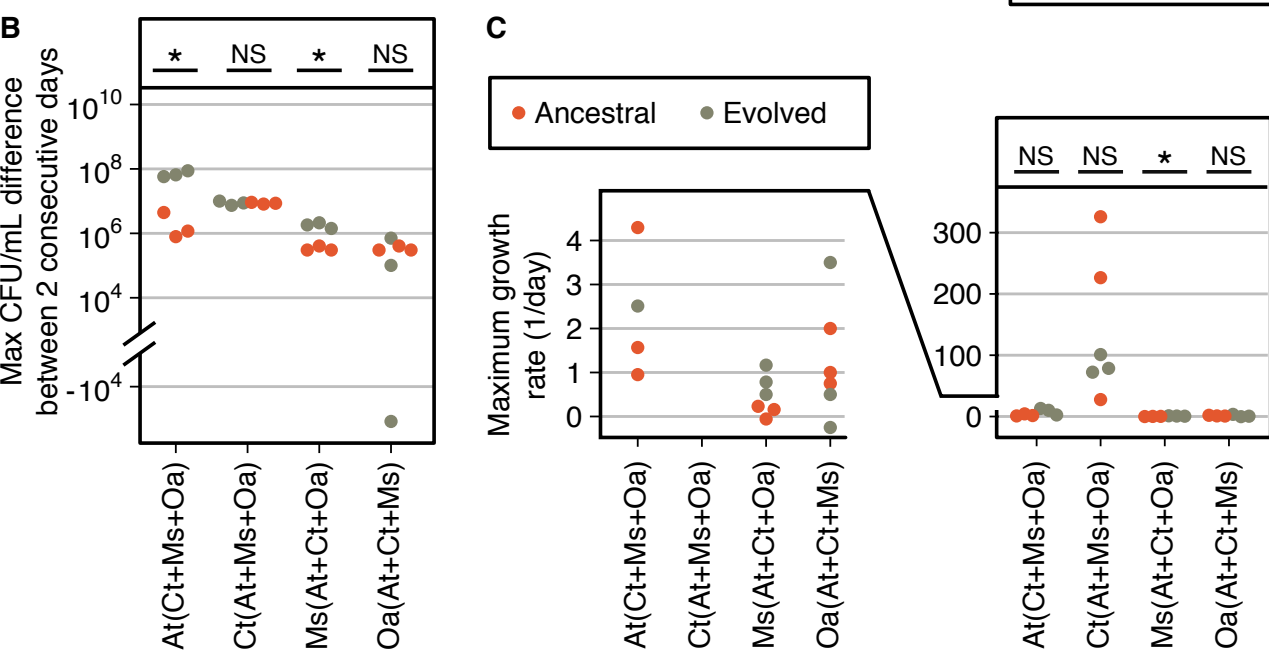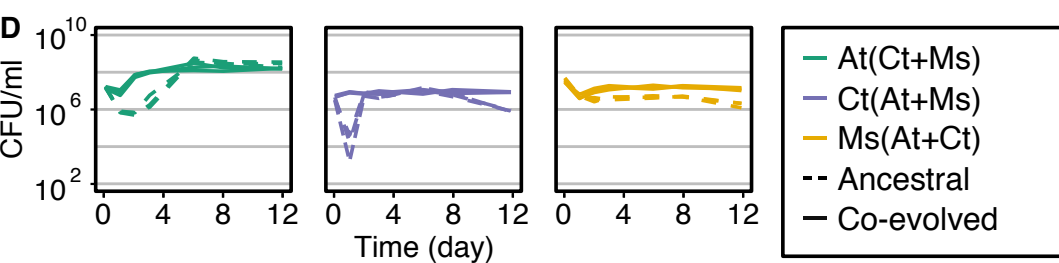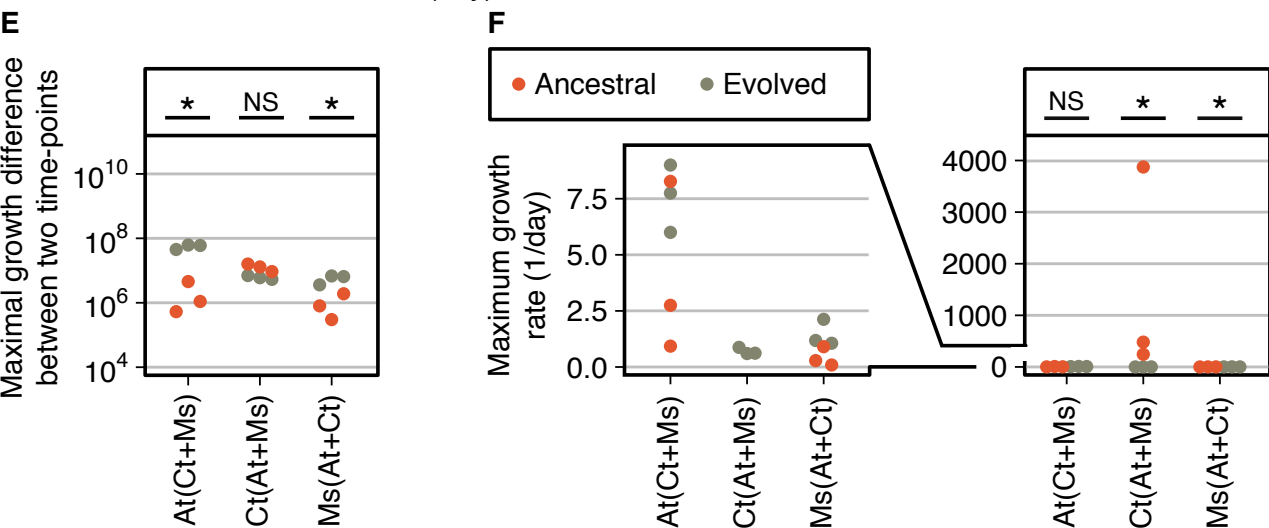

Supplement: Supplementary file 6 — Figure S5 [file 41396_2022_1314_MOESM6_ESM.pdf]

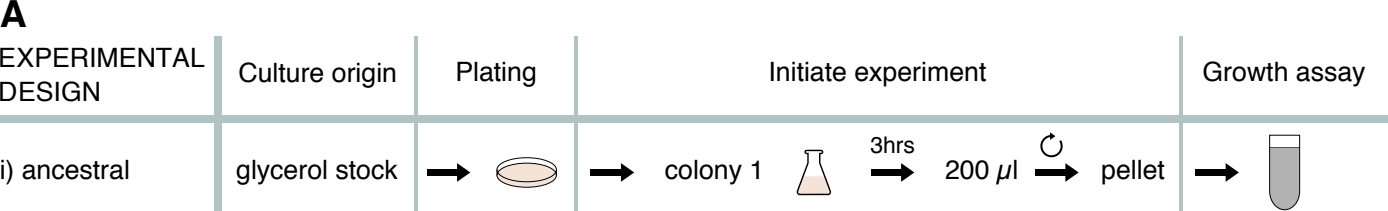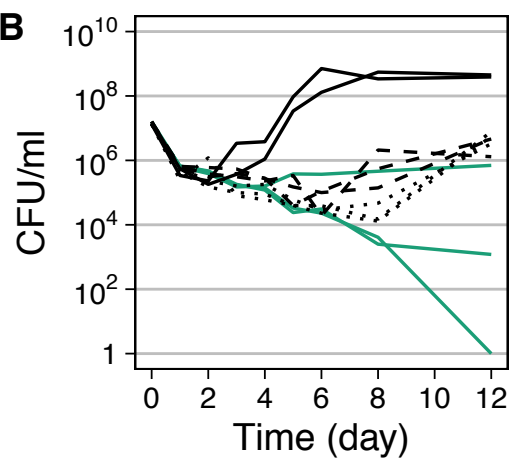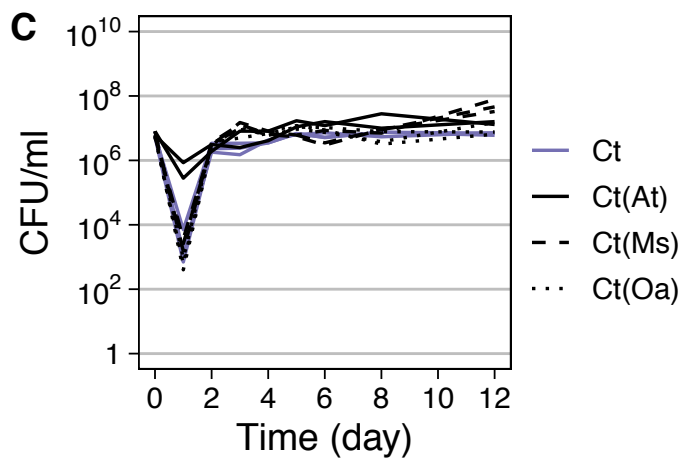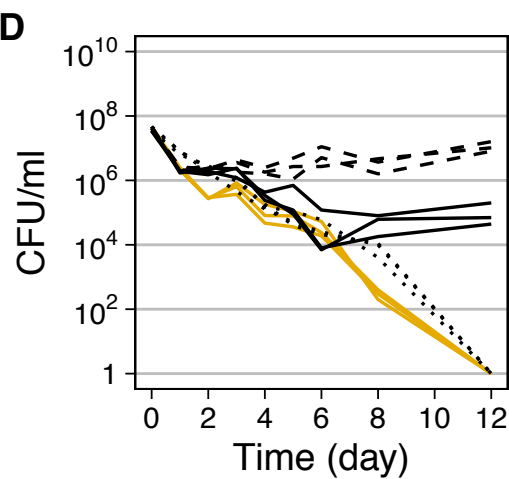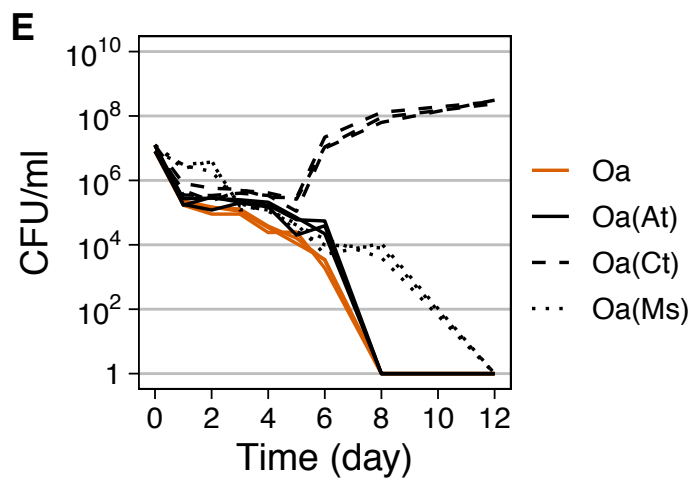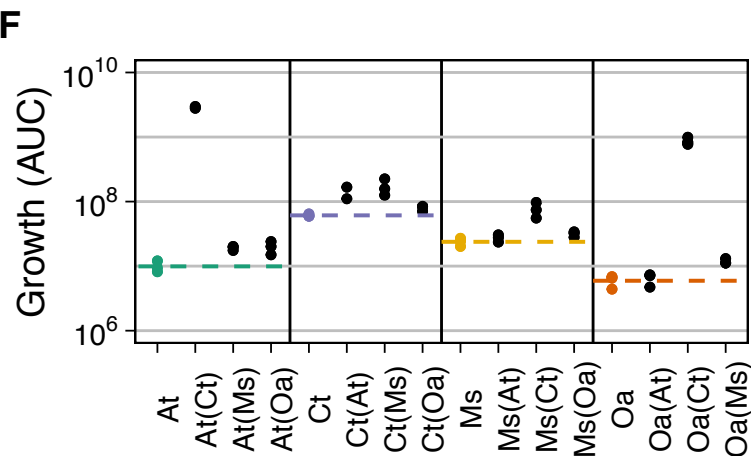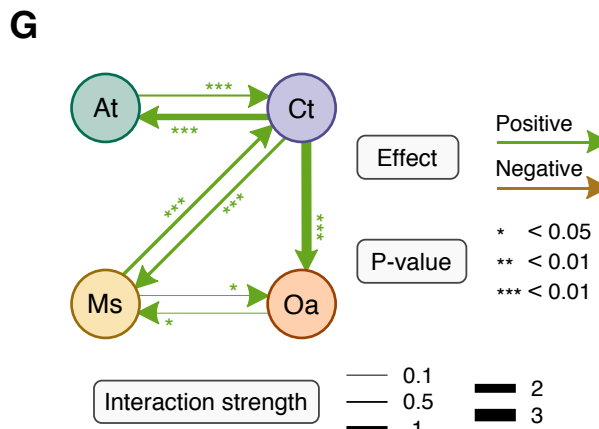

Supplement: Supplementary file 7 — Figure S6 [file 41396_2022_1314_MOESM7_ESM.pdf]

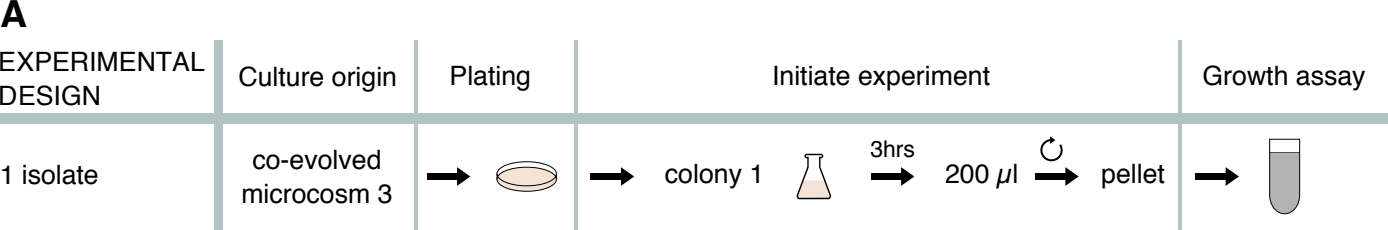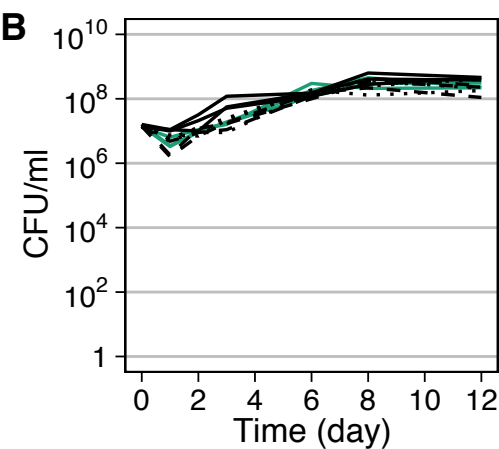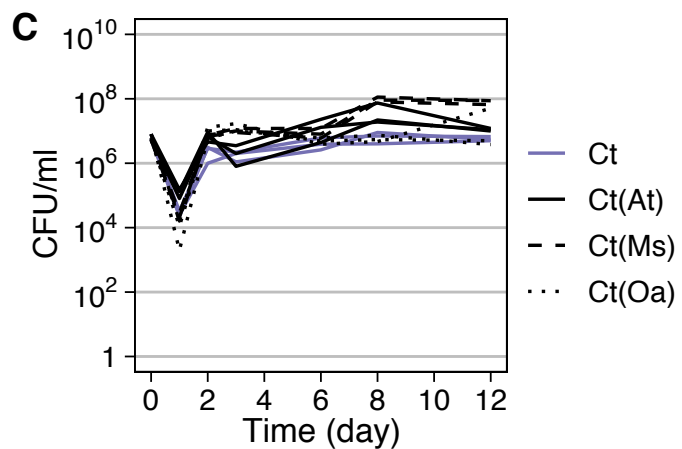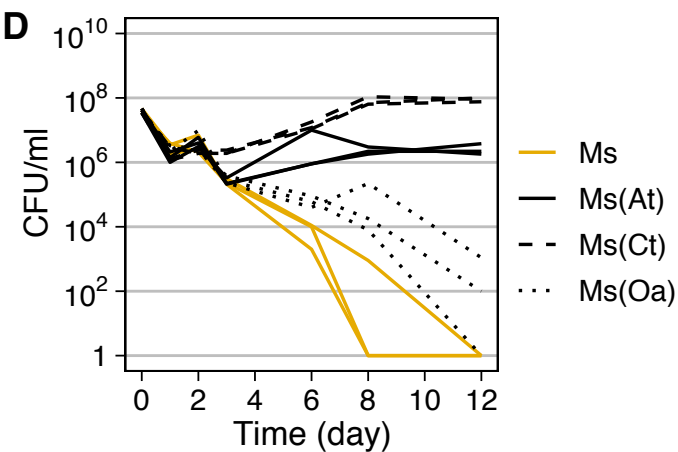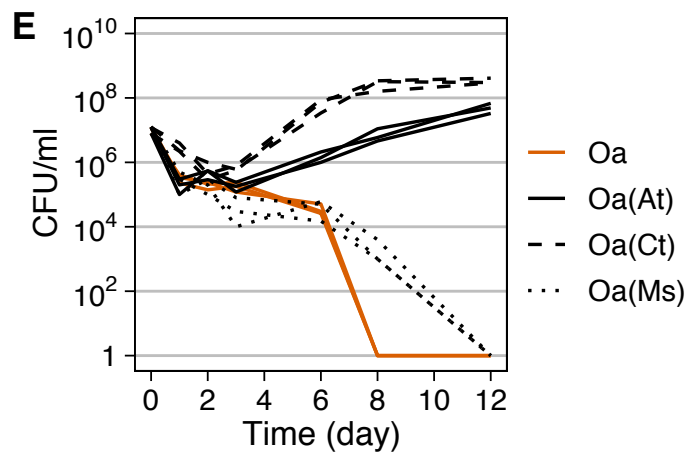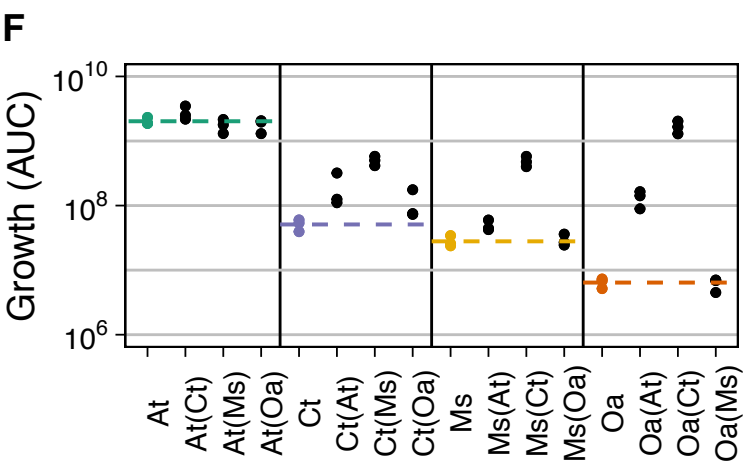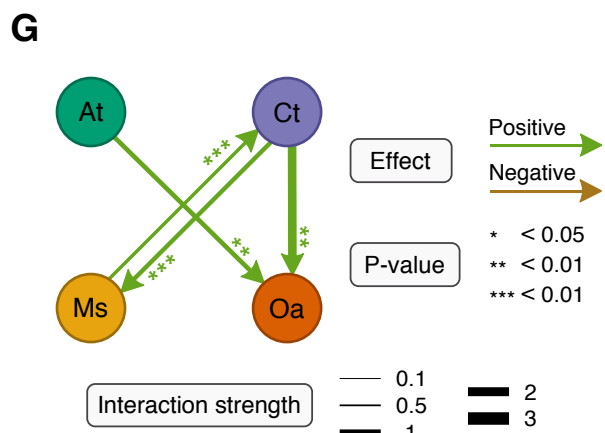

Supplement: Supplementary file 8 — Figure S7 [file 41396_2022_1314_MOESM8_ESM.pdf]

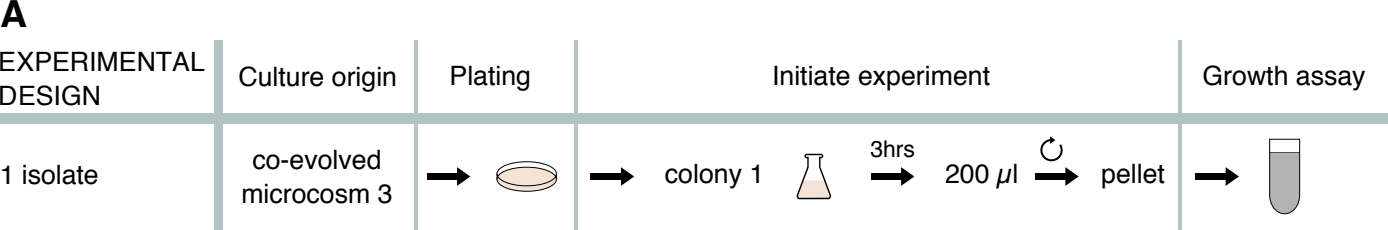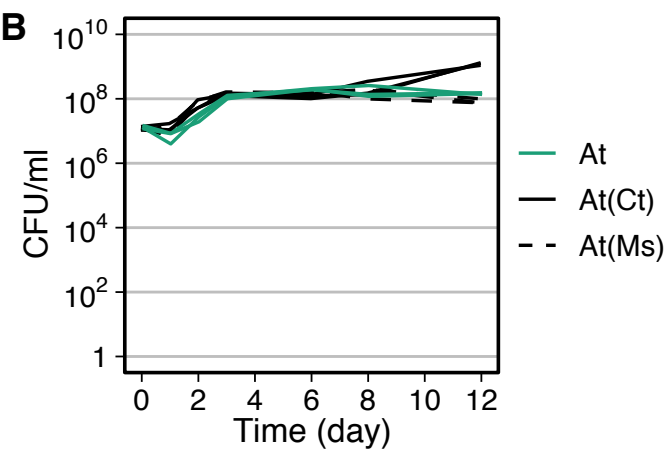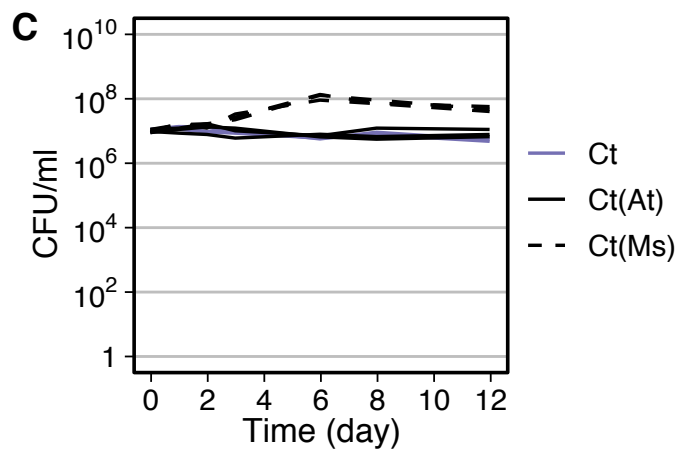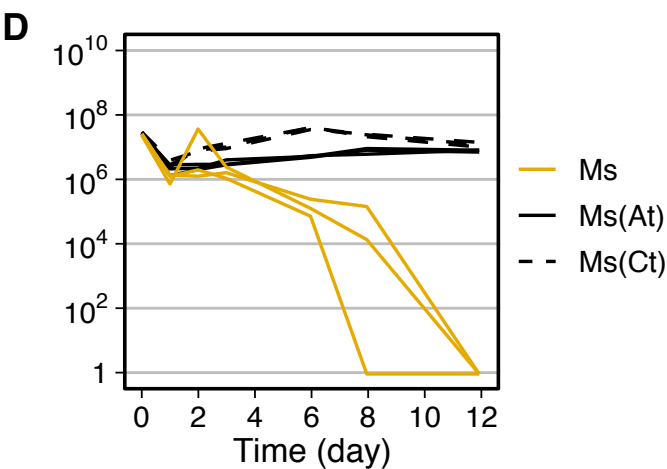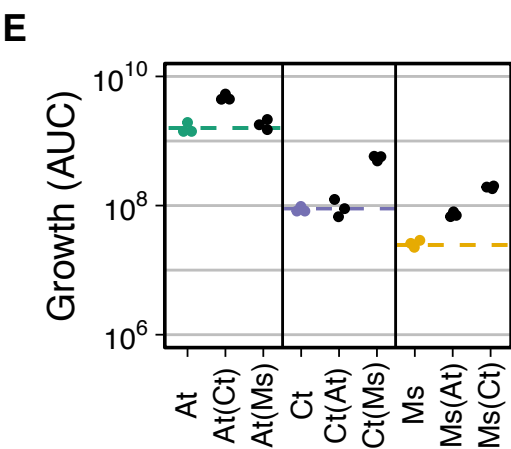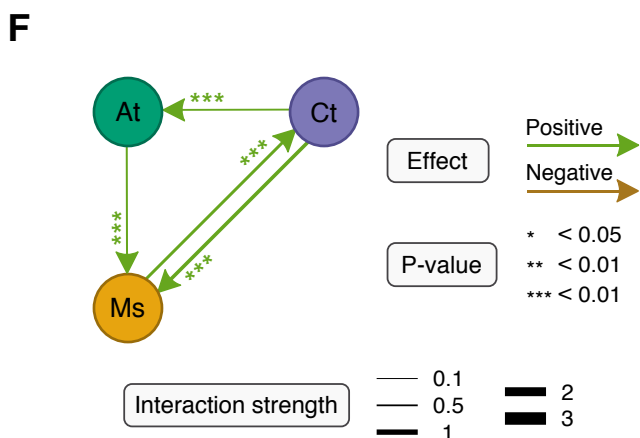

Supplement: Supplementary file 9 — Figure S8 [file 41396_2022_1314_MOESM9_ESM.pdf]

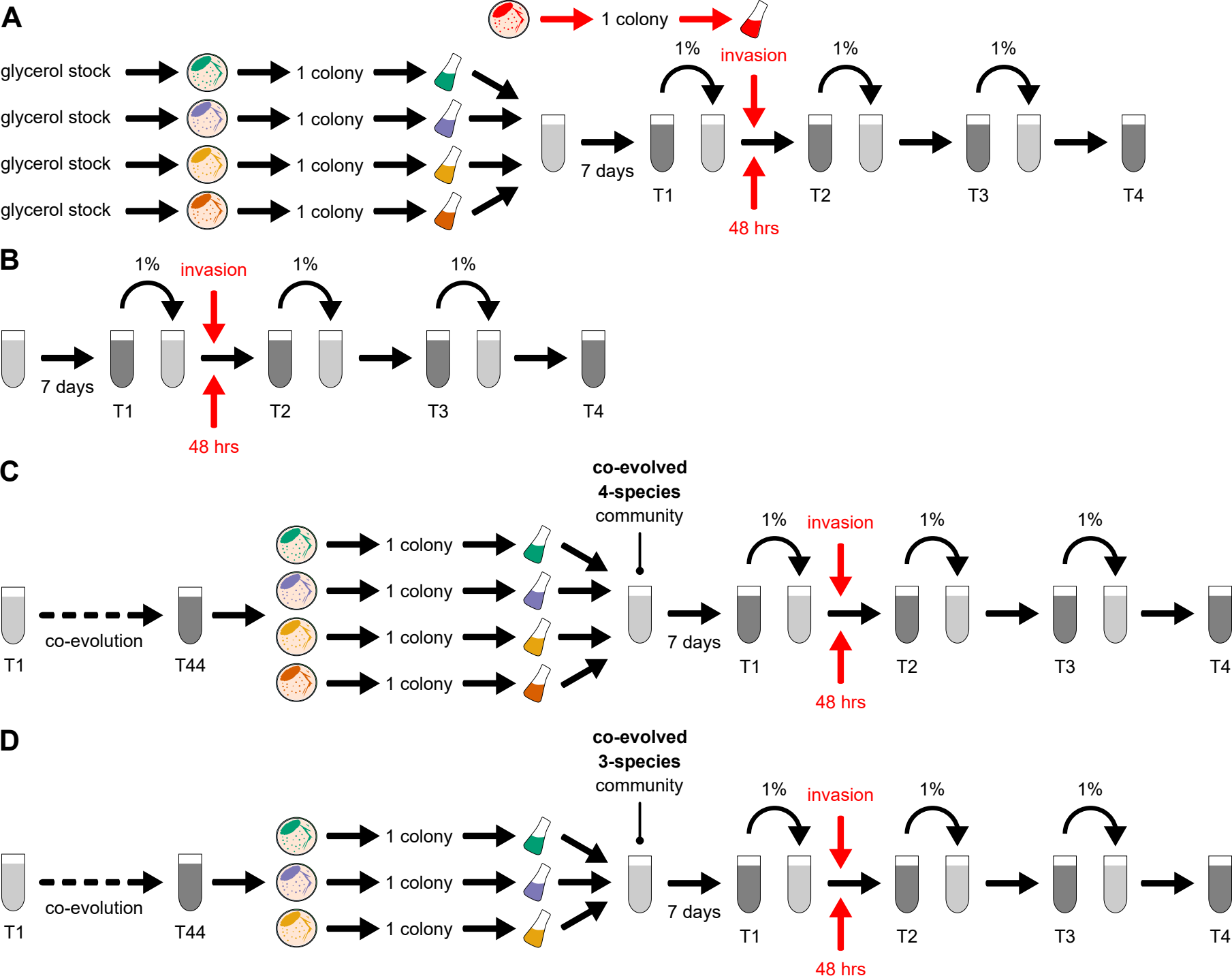

### Legend

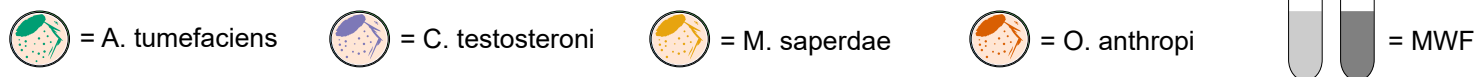

T1, T2, ..., Tn = Transfer 1, Transfer 2, ... Transfer n

Supplement: Supplementary file 10 — Figure S9 [file 41396_2022_1314_MOESM10_ESM.pdf]

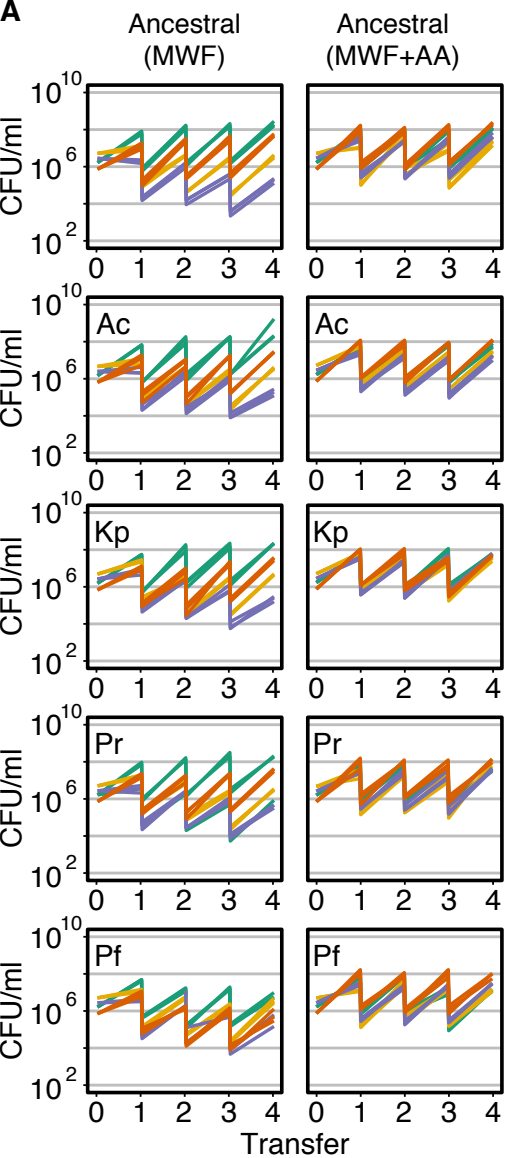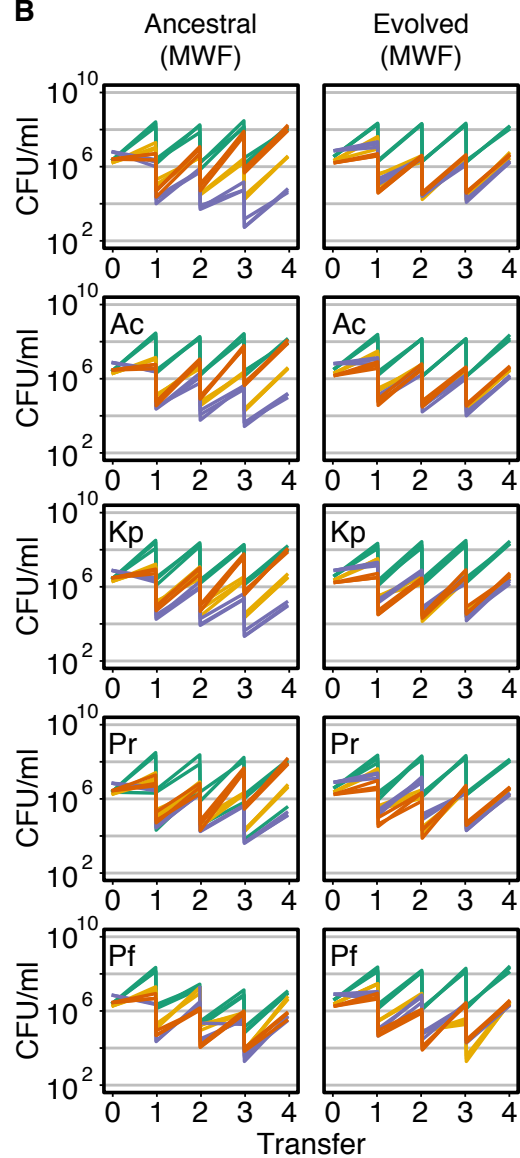

— At — Ct — Ms — Oa

Supplement: Supplementary file 11 — Figure S10 [file 41396_2022_1314_MOESM11_ESM.pdf]

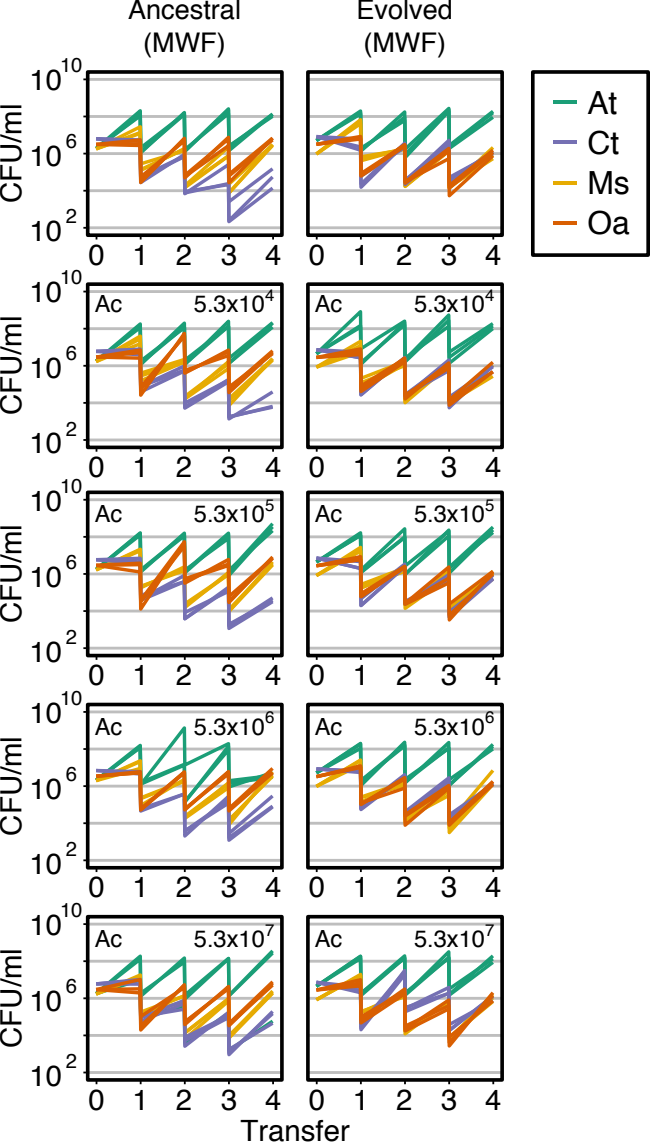

Supplement: Supplementary file 12 — Figure S11 [file 41396_2022_1314_MOESM12_ESM.pdf]

Ancestral  
(MWF)

Evolved  
(MWF)

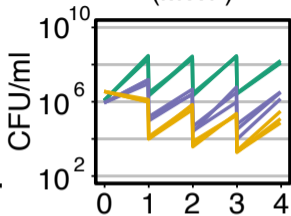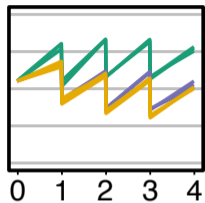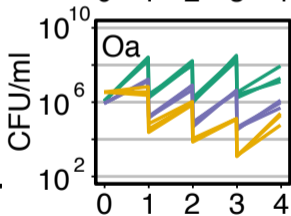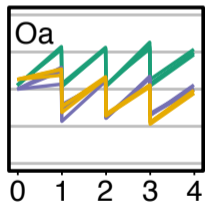

Transfer

At Ct Ms

Supplement: Supplementary file 13 — Figure S12 [file 41396_2022_1314_MOESM13_ESM.pdf]

**A**

CFU/ml at transfer 4

**MWF**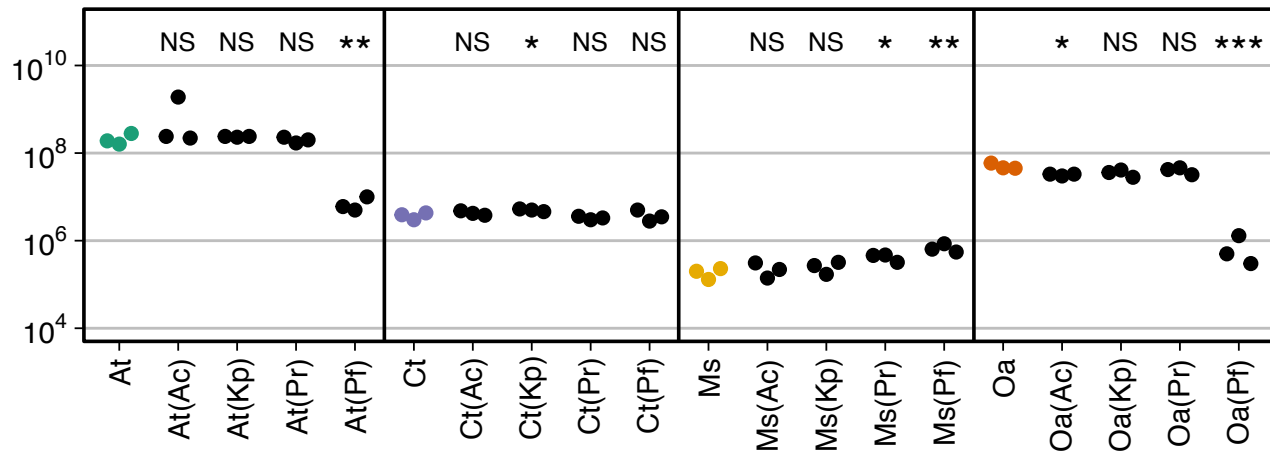**B**

CFU/ml at transfer 4

**MWF+AA**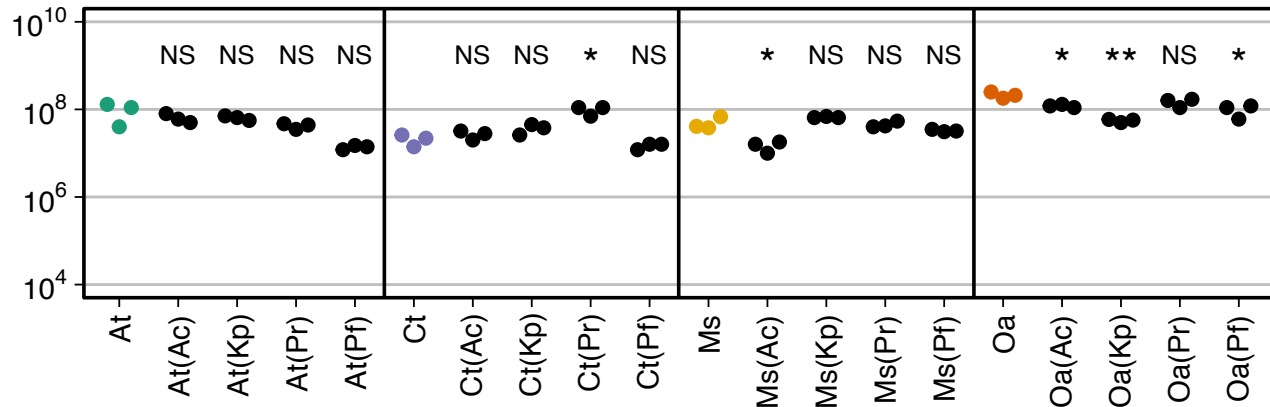

Supplement: Supplementary file 14 — Figure S13 [file 41396_2022_1314_MOESM14_ESM.pdf]
